# Targeted next generation sequencing revealed a novel deletion-frameshift mutation of *KCNH2* gene in a Chinese Han family with long QT syndrome: A case report and review of Chinese cases

**DOI:** 10.1097/MD.0000000000019749

**Published:** 2020-04-17

**Authors:** Fengli Du, Guangxin Wang, Dawei Wang, Guoying Su, Guixiang Yao, Wei Zhang, Guohai Su

**Affiliations:** a Institute of Translational Medicine, Jinan Central Hospital Affiliated to Shandong University; b Department of Postgraduate, Shandong First Medical University, Jinan, Shandong; c Department of Biomedical Sciences, City University of Hong Kong, Hong Kong SAR, China; d Department of Cardiology, Jinan Central Hospital Affiliated to Shandong University, Jinan, Shandong, China.

**Keywords:** *KCNH2* gene, long QT syndrome, LQT2, mutation, next-generation sequencing

## Abstract

**Introduction:**

Long QT syndrome (LQTS) is electrocardiographically characterized by a prolonged QT interval and manifests predisposition to life-threatening arrhythmia which often leads to sudden cardiac death. Type 2 LQTS (LQT2) is the second most common subtype of LQTS and caused by mutations in *KCNH2* gene. Up to date, >900 mutations have been reported to be related to LQT2. However, mutational screening of the *KCNH2* gene is still far from completeness. Identification of *KCNH2* mutations is particularly important in diagnosis of LQT2 and will gain more insights into the molecular basis for the pathogenesis of LQT2.

**Patient concerns:**

A Chinese Han family with LQTS phenotypes was examined.

**Diagnosis:**

A novel deletion-frameshift mutation, c.381_408delCAATTTCGAGGTGGTGATGGAGAAGGAC, in exon 3 of *KCNH2* gene was identified in a Chinese family with LQTS. On the basis of this finding and clinical manifestations, the final diagnosis of LQT2 was made.

**Interventions:**

Next-generation sequencing (NGS) of DNA samples was performed to detect the mutation in the LQTS-related genes on the proband and her mother, which was confirmed by Sanger sequencing. The proband was then implanted with an implantable cardioverter defibrillator and prescribed metoprolol 47.5 mg per day.

**Outcomes:**

This novel heterozygous mutation results in a frameshift mutation after the 128^th^ residue (Asparagine), which replaced the original 1031 amino acids with 27 novel amino acids (p.N128fsX156).

**Conclusion:**

This novel mutation presumably resulted in a frameshift mutation, p.N128fsX156. Our data expanded the mutation spectrum of *KCNH2* gene and facilitated clinic diagnosis and genetic counseling for this family with LQTS.

## 1 Introduction

Long QT syndrome (LQTS) is a cardiovascular disorder characterized by prolonged QT interval on ECG and presence of syncope, seizures, and sudden death with an incidence of about 1 in 2500.^[1,2]^ Genetic studies have so far identified 15 subtypes of LQTS (LQT1_LQT15) caused by mutations in genes of cardiac ion channels or ion channel modulators, including membrane adapters. Type 2 LQTS (LQT2, OMIM #613688) is reported to be the second most common form of LQTS and accounts for approximately 30% of mutation-positive LQTS.^[3,4]^

LQT2 is inherited in autosomal dominant manner and caused by mutations of potassium voltage- gated channel subfamily H member 2 (*KCNH2*) on chromosome 7q36.1. Up to date, >900 mutations have been reported to be related to LQT2 according to the Human Gene Mutation Database (HGMD, http://www.hgmd. org/), Pubmed, Embase, and Web of science. However, mutational screening of the *KCNH2* gene is still far from completeness. Identifying more novel mutations will gain more insights into the molecular basis for the pathogenesis of LQT2.

Genetic studies are essential for the diagnosis, prognosis and treatment of genetic diseases. With the development of new sequence technology, next-generation sequencing (NGS) has recently been used as an alternative approach to more traditional methods in the clinical practice including genetic diagnosis, family genetics counseling, and prenatal diagnostic testing. NGS has many advantages which can not only to produce massive amounts of data in parallel but also to measure each base pair to an unprecedented depth, which greatly reduces the time and cost of sequencing each sample at each locus.^[5]^

In this study, we described the clinical and ECG manifestations of a Chinese Han family with LQTS. Then, we used a method based on targeted gene capture and next-generation sequencing in this family and identified a novel deletion mutation, c.381_408delCAATTTCGAGGTGGTGATGGAGAAGGAC, in exon 3 of *KCNH2* gene. Identification of this novel mutation provides new insights into the molecular basis for the pathogenesis of LQTS and assists early diagnosis.

## 2 Patients and methods

### 2.1 Proband and family investigation

**Figure 1:**
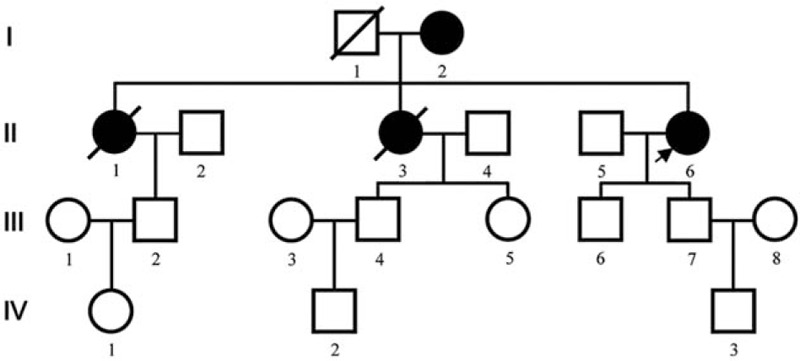
Pedigree of a Chinese Han family with LQTS (The arrow indicates the proband). LQTS = long QT syndrome.

Figure 1 shows the pedigree of the LQTS family. The proband (II-6) was a 47 year old Chinese Han female, who was born at full term after an uncomplicated pregnancy and delivery. She was referred to Jinan Central Hospital affiliated to Shandong University on September 28, 2018 because of her syncope an hour ago. She had experienced 10 episodes of syncope before 10 years and had not taken any medication for it. Her mother also had the symptom of syncope. Family history investigation revealed that her 2 sisters had unexplained syncope and died suddenly at the age of 42 and 20 years, respectively.

All available individuals with or without a positive history underwent a full physical examination, including QT interval assessment and T-wave morphology through ECG. The QT intervals were measured by Ashman method and corrected for heart rate, that is rate-corrected QT (QTc).^[1]^ The family members were clinically diagnosed with LQTS if they had a prolonged QT interval (QTc ≥470 ms for male; QTc ≥480 ms for female), a history of syncope, cardiac arrest, or sudden death.^[6]^

This study was approved by the research ethics committee of Jinan Central Hospital Affiliated to Shandong University. Informed consents were obtained from all subjects or their legal guardians. The proband have provided informed consent for publication of the case.

### 2.2 Methods

#### 2.2.1 Targeted sequence capture and NGS

NGS was performed on the proband and her mother (II-6; I-2).

Two microliters of peripheral blood were collected and then preserved in K_2_-EDTA tubes. Genomic DNA was isolated from peripheral whole blood using TIANamp Blood DNA Kit (Tiangen Biotech Beijing Co. LTD., China). After the DNA extraction, target sequences were enriched by using customized capture probes chips (Illumina, San Diego), which included 15 genes (*KCNQ1*, *KCNH2*, *SCN5A*, *ANK2*, *KCNE1*, *KCNE2*, *KCNJ2*, *CACNA1C*, *CAV3, SCN4B, AKAP9, SNTA1, KCNJ5, CALM1* and *CALM2* gene) that are associated with LQTS. DNA probes were designed for exons and flanking intron sequences (−20 base pairs). One microgram genomic DNA was fragmented into 200 to 300 bp length by Covaris Acousitic System. The DNA fragments were then processed by end-repairing, A-tailing and adaptor ligation, a 4-cycle pre-capture PCR amplification, targeted sequences capture. Captured DNA fragments were eluted and amplified by 15 cycle post capture PCR. The final products were sequenced with 150-bp paired-end reads on Illumina HiSeq X Ten platform according to the standard manual.

The clean short-reads were mapped to human genome (hg19) using BWA software (http:// sourceforge.net/projects/bio-bwa/). SAM tools Pileup software (http://sourceforge.net/projects/samtools/) and SOAP snp software (http://soap.genomics.org.cn/) were used to detect single nucleotide variants (SNPs) and small insertions and deletions. Mutations were annotated by ANNOVAR software, which is freely available at http://www.openbioinformatics.org/annovar/. Mutations were interpreted according to the American College of Medical Genetics and Genomics (ACMG) recommended standard.^[7]^

#### 2.2.2 Sanger sequencing

To validate true positive novel mutations identified by NGS, Sanger sequencing was performed to confirm the presence or absence of these mutations in the proband, other living affected family member, unaffected family members and 50 unrelated healthy controls.

The specific PCR primers (forward primer 5’-ATGTGGTGCCCGTGAAGAAC-3’, reverse primer 5’-GGGAAGCCCAACAGATGAAG-3’) were used for the amplification of exon 3 in *KCNH2* gene based on the reference sequences of human genome from GenBank in NCBI ( NC_000007.14). PCR cycling was performed on a DNA thermal cycler (Gene Amp 9700, Perkin-Elmer, USA) with 2×Hotstart Taq PCR Mastermix kit (Tiangen Biotech Beijing Co. LTD., China). In a 50 μL reaction mix, 300 ng of genomic DNA were used with 2.0 μL of each primer (10 μmol/L), and 25 μL of 2×PCR Mastermix. Genomic DNA was first denatured at 94°C for 3 minutes, followed by 31 cycles of 94°C for 35 seconds, 59.5°C for 35 seconds, and 72°C for 50 seconds. The PCR products were extended at 72°C for 5 minutes. The products were gel-purified with an agrose gel DNA purification kit (Tiangen Biotech Beijing Co. LTD., China), and the purified PCR products were sequenced using the forward and reverse primers. Automated sequencing was performed at both ends on an ABI 377 automatic sequencer.

## 3 Results

### 3.1 Clinical data

**Figure 2:**
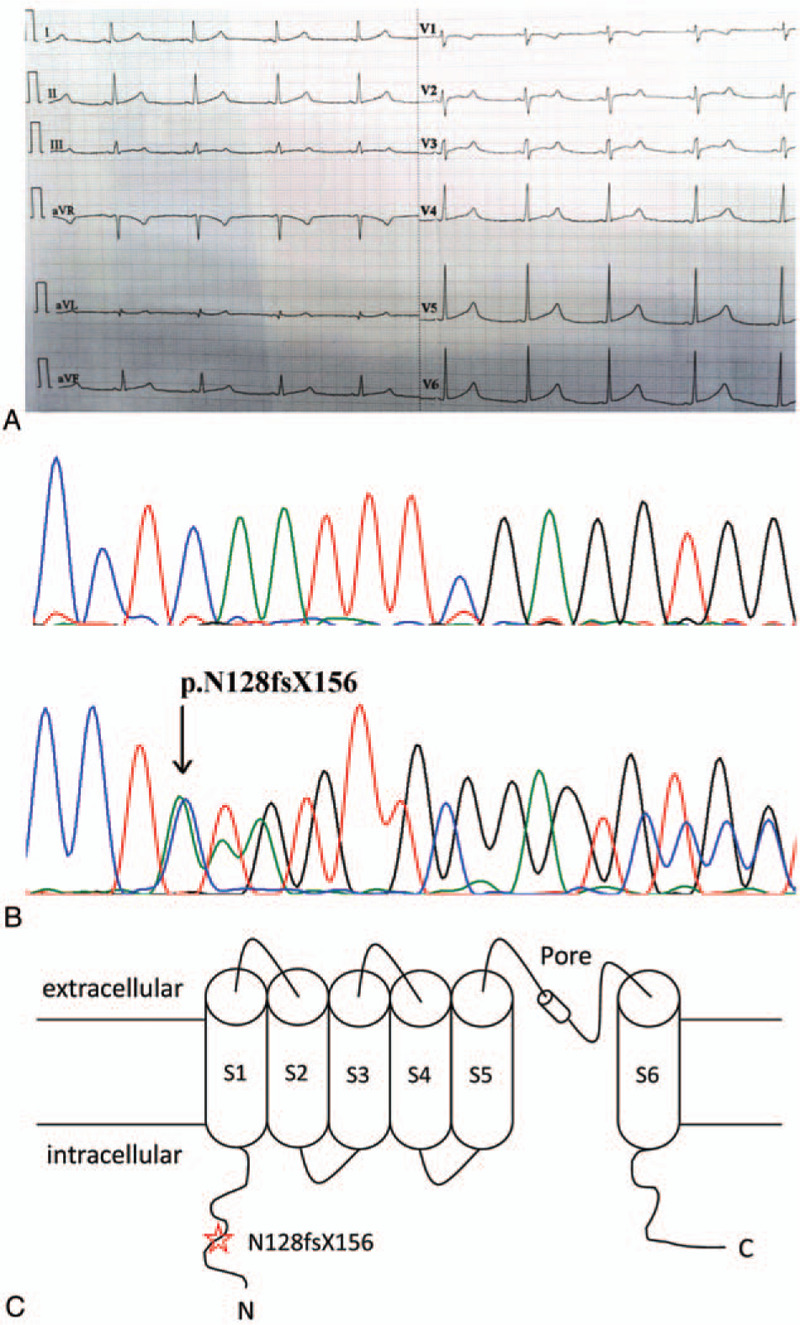
ECG and mutation analysis. (A) ECG of the proband. The resting 12-lead ECG revealed the prolonged QT interval and the subtle bifid T wave (V5; V6). (B) Sanger sequencing chromatogram showing a heterozygous deletion mutation, c.381_408delCAATTT CGAGGTGGTGATGGAGAAGGAC in *KCNH2* gene of the proband (lower Sanger sequencing chromatogram) and Sanger sequencing chromatogram in a healthy individual (upper Sanger sequencing chromatogram). The arrow indicates the mutation site. (C) A schematic topology showing the structure domains of Kv11.1 and locations of the p.N128fsX156 described.

12-lead ECG of the proband showed a prolonged QTc interval of 481 ms and a subtle bifid T wave with second component on top of T wave in lead V5 and V6 (Fig. 2AA). Her physical examination showed normal BP (117/68 mmHg). There were no other abnormal physical findings and no structural cardiac abnormalities on the ultrasound cardiography (UCG). The clinical characteristics of most of living family member are summarized in Table 1.

**Table 1:**
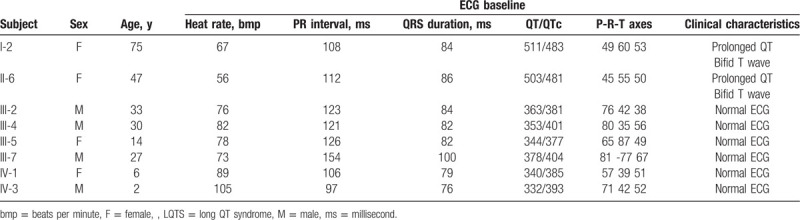
Clinical characteristics of most of living members in a Chinese Han family with LQTS.

The proband was clinically diagnosed as LQTS. She was then implanted with an ICD and prescribed metoprolol 47.5 mg/day. Neither arrhythmia nor syncope has been observed during the 11-month follow-up period.

### 3.2 Mutation detection

In this study, 85 variants in 15 genes associated with LQTS were detected by next-generation sequencing. We then excluded those variants with an allele frequency >5% in the dbSNP data-base, 1000 human genome dataset, exome aggregation consortium (ExAC), and genome aggregation database (gnomAD). According to the detailed filtering criteria and analysis pipeline published before,^[5]^ a deletion mutation, c.381_408delCAATTTCGAGGTGGTGATGGAGAAGGAC in exon 3 of *KCNH2* gene, was revealed in both the proband and her mother. This mutation was then confirmed by Sanger sequencing (Figure 2 B). The heterozygous mutation resides in the N-terminus of Kv11.1 (Fig. 2C) and results in a frameshift mutation after the 128^th^ residue (Asparagine), which replaces the original 1031 amino acids with 27 novel amino acids (p.N128fsX156). No mutation at this site was found in available unaffected family members or in 50 unaffected, unrelated healthy controls. According to the HGMD (http://www.hgmd.cf.ac.uk/docs/login. html), this heterozygous mutation is novel.

## 4 Discussion

LQTS patients are often first sent to the hospital after episodes of syncope and/or seizure, and their ECG reveals a prolonged QT interval. The diagnosis of LQTS is suspected on occurrence of syncope or cardiac arrest, and prolonged QTc which is generally referenced as >470 ms for males and >480 ms for females on ECG.^[6,8]^ In the present study, a Chinese Han family with LQTS is reported. All patients have typical features of the LQTS, and QTc in the resting 12-lead ECG of living affected members is more than 480 ms, which are consistent with criteria for LQTS proposed by Schwartz et al.^[9]^ Furthermore, by using NGS and Sanger sequencing, we identified a novel deletion-frameshift mutation, c.381_408delCAATTT CGAGGTGGTGATGGAGAAGGAC, in exon 3 of *KCNH2* gene in this Chinese family. On the basis of above genetic finding and clinical manifestations, the final diagnosis of LQT2 was made in this family.

Human *KCNH2* gene was identified as the LQT2 gene in 1995. This gene consists of 15 exons and 14 introns, and encodes the voltage-gated K^+^ (Kv) channel α-subunit Kv11.1, which is also called the human ether a go-go-related K^+^ channel protein. Kv11.1, an 1159 amino acid polypeptide, comprises 6-helical transmembrane domains (S1 to S6) involving amino acid residues from 398 through 657, a K^+^-selective pore domain situated between S5 and S6 (S5-pore-S6 region: 552 to 657), and *N*- terminus before residue 398 and C-terminus after residue 657.^[10]^ This protein is expressed mainly in heart muscle, and 4 Kv11.1 α-subunits co-assemble into a tetrameric ion channel that conducts the rapidly activating delayed rectifier K^+^ current (I_Kr_) in the heart, which plays an essential role in the final repolarization of the ventricular action potential.^[4]^ The deletion-frameshift mutation p.N128fsX156 revealed in this study, generates a stop codon at 156 unexpectedly and can truncate the protein, losing most part of N- terminus, all of 6-helical transmembrane domains, the pore domain and C-terminus, which presumably leads to deficiency in I_Kr_ and contributes to lengthening of the QT interval.^[11]^

Up to date, >900 mutations have been reported in the *KCNH2* gene for LQT2 according to HGMD, Pubmed, Embase, and Web of Science. Those mutations include missense mutation, frameshift mutation due to deletion or insertion, nonsense mutation, splice error mutation, and repeat variations. The literature review of Chinese cases suggests that 24 different *KCNH2* sequence variants have been reported in Chinese LQT2 patients, the most common being missense mutations (Table 2).^[12–31]^ These mutations of *KCNH2* were frequently located in S5 domain and C-terminus. p.N128fsX156 founded in our research, which resides in the N-terminus of Kv11.1, is the third deletion-frameshift mutation in Chinese cases. After searching the SNP database and the human gene mutation database, we found that p.N128fsX156 mutation was absent from these databases. We demonstrate that this family carries a novel heterozygous mutation based on the following evidences. First, the mutation is not present in the unaffected family members or in 50 unrelated healthy controls. Second, the mutation truncates the protein, losing all transmembrane domains and the pore domain.

**Table 2:**
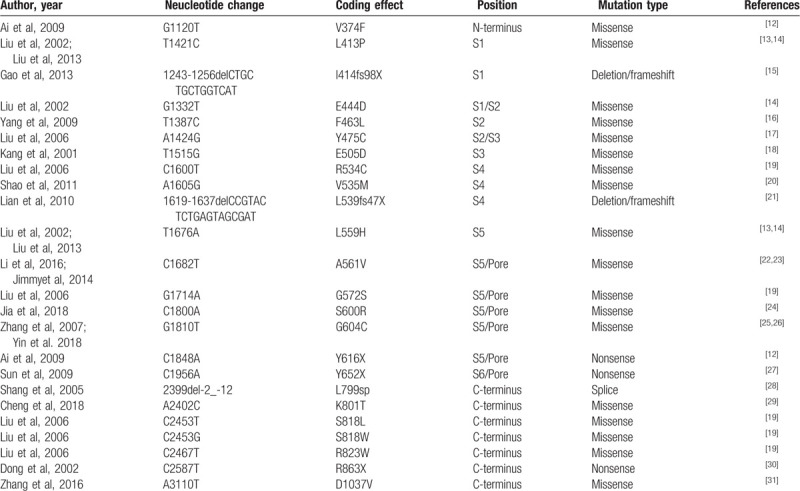
The summary of previously reported *KCNH2* gene mutations in Chinese mainland.

In a previous study of patients with LQT2, mutations in pore region were associated with an increased risk for arrhythmia-related cardiac events when compared to patients with non-pore mutations.^[32]^ However, our study was limited by population size in its ability to explore the genotype-phenotype effect within distinct domain of the non-pore regions.

## 5 Conclusion

In summary, we present evidence that p.N128fsX156 mutation represents a novel *KCNH2* mutation in a Chinese Han family with LQTS. This novel loss of function mutation truncates the protein. Our data extend the mutation spectrum of *KCNH2* gene and facilitate clinic diagnosis and genetic counseling for this family with LQT2.

## Acknowledgments

The authors thank the patients and their family members for their cooperation in this study.

## Author contributions

**Data curation:** Fengli Du, Guangxin Wang.

**Formal analysis:** Fengli Du, Guangxin Wang.

**Funding acquisition:** Guohai Su, Wei Zhang.

**Investigation:** Fengli Du, Guangxin Wang, Guoying Su, Guixiang Yao.

**Project administration:** Guangxin Wang, Dawei Wang, Guohai Su.

**Resources:** Guangxin Wang, Dawei Wang, Wei Zhang.

**Software:** Dawei Wang, Guoying Su.

**Supervision:** Guohai Su, Wei Zhang.

**Writing – original draft:** Fengli Du, Guangxin Wang.

**Writing – review & editing:** Guohai Su, Wei Zhang.
